# Blastic plasmacytoid dendritic cell neoplasm with leukemic manifestation and ETV6 gene rearrangement: A case report

**DOI:** 10.3892/etm.2015.2236

**Published:** 2015-01-29

**Authors:** NA GAO, XUE-XIA WANG, JIAN-RONG SUN, WEN-ZHENG YU, NONG-JIAN GUO

**Affiliations:** 1 Department of Hematology, Binzhou Medical University Hospital, Binzhou, Shandong 256603, P.R. China; 2 Department of Hematology, Central Hospital of Jinan, Shandong University School of Medicine, Jinan, Shandong 250013, P.R. China

**Keywords:** blastic plasmacytoid dendritic cell neoplasm, ETS variant 6 gene, leukemic manifestation, CD56

## Abstract

Blastic plasmacytoid dendritic cell neoplasm (BPDCN) is a rare malignant tumor of the hemopoietic system that arises from plasmacytoid dendritic cell precursors with a highly aggressive course. BPDCN frequently involves the skin, lymph nodes, peripheral blood and bone marrow. BPDCN is known to develop leukemic dissemination as a feature of myelomonocytic leukemia in the late phase of the disease, which leads to a poorer prognosis. In the present study, a case of BPDCN with leukemic manifestation without cutaneous involvement was reported. In addition, ETS variant gene 6 (ETV6) gene rearrangement was detected in the patient. The patient relapsed soon after complete remisson and had no response to further treatment. To the best of our knowledge, this is the first reported case of BPDCN with ETV6 rearrangement. Following chemotherapy treatment, the patient suffered from severe headache in the complete remission stage; however, brain CT scans showed no significant abnormalities. Several lumbar punctures and intrathecal chemotherapy were performed, and the patient recovered gradually. Therefore, the patient was considered to suffer from central nervous system leukemia. In conclusion, implementation of lumbar punctures and preventive intrathecal chemotherapy are required in BPDCN patients with leukemic manifestation during the remission stage.

## Introduction

Blastic plasmacytoid dendritic cell neoplasm (BPDCN) is a rare neoplasm, previously termed as blastic natural killer (NK)-cell lymphoma. BPDCN is included in the acute myeloid leukemia (AML) category of the latest World Health Organization (WHO) classification of tumors (1). BPDCN often involves the skin, lymph nodes, peripheral blood and bone marrow. Blast cells are generally found to be positive for CD4, CD56 and CD123 in patients with BPDCN (2). The disease is not sensitive to traditional chemotherapy treatment, and the median survival time is ~12–14 months (1). ETS variant gene 6 (ETV6) gene-involved chromosomal translocations have been observed in numerous hematological malignancies (3). Several studies exist on BPDCN; however, to the best of our knowledge, BPDCN with ETV6 rearrangement has not been previously reported. Initially, BPDCN usually affects the skin and then involves the lymph nodes and bone marrow, ultimately proliferating in the peripheral blood (4,5). BPDCN has numerous characteristics that are similar to leukemia. Thus, lumbar punctures and preventive intrathecal chemotherapy are indispensable measures for the treatment of acute leukemia patients in the remission stage. Lumbar punctures and preventive intrathecal chemotherapy may be required in BPDCN patients with leukemia manifestation during the remisson stage. In the present study, a case of BPDCN with leukemic manifestation without cutaneous involvement was reported.

## Case report

**Figure 1:**
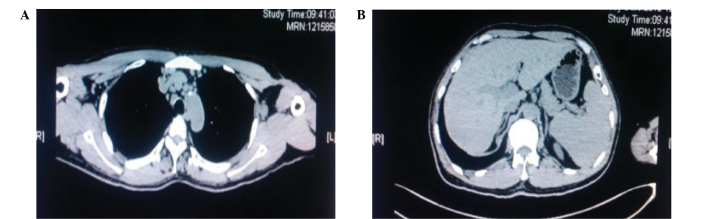
Computed tomography scans showing multiple (A) mediastinal and (B) peritoneal lymphadenopathies.

A 48-year-old male, who was previously healthy, was admitted to the Affiliated Hospital of Binzhou Medical University Hospital (Binzhou, China) in December 2012, due to experiencing left cervical lymphadenopathy for two months. Physical examination revealed a number of superficial lymphadenopathies and splenomegaly. The patient did not present hepatomegaly or skin lesions. In a preliminary blood test, the patient was found to have a hemoglobin level of 12.8 g/dl, white blood cell count of 2.8×10^9^/l, platelet count of 129×10^9^/l and erythrocyte sedimentation rate of 33 mm/h. No abnormalities were observed in the liver function and biochemical assays, while blood coagulation and urine tests were found to be unremarkable. In addition, a human immunodeficiency virus antibody test was negative. Computed tomography (CT) scans of the chest and abdomen revealed splenomegaly and multiple deep lymphadenopathies (Fig. 1).

**Figure 2:**
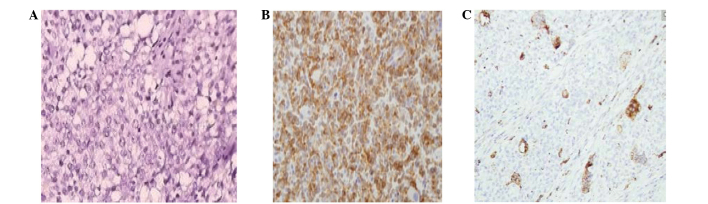
Immunohistological staining of lymph nodes revealed that the cells were positive for (A) CD43, (B) CD123 and (C) CD68 (magnification, ×100).

**Figure 3:**
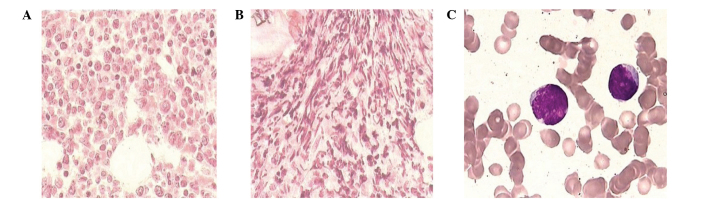
Bone marrow biopsy revealed (A) abnormal diffuse infiltration of tumor cells and (B) collagen fibrous tissue hyperplasia (hematoxylin and eosin staining; magnification, ×400). (C) Tumor cells of various sizes with basophilic cytoplasm and irregular nuclei were observed in the peripheral blood (Wright-Giemsa staining; magnification, ×1,000).

**Figure 4:**
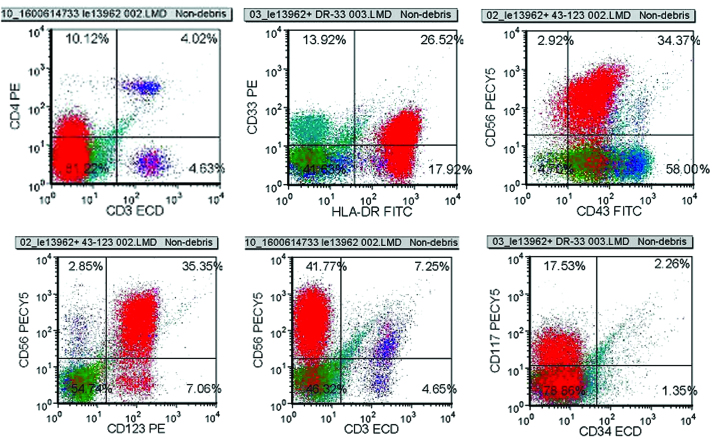
Flow cytometric analysis revealed that abnormal bone marrow cells were positive for CD33, CD117, CD56, CD4 and CD123. The red signals indicate the abnormal cells.

**Figure 5:**
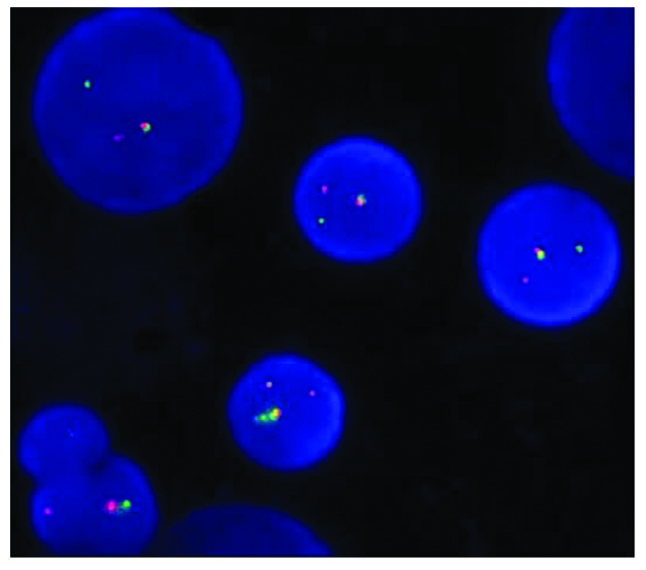
Split-signal fluorescence *in situ* hybridization analysis of ETV6 rearrangement in a patient with blastic plasmacytoid dendritic cell neoplasm. The red and green fluorescence signal separation shows the presence of ETV6 gene rearrangement. Red and green fluorescence signal separation was observed in the cell nuclei using fluorescence microscopy. Magnification, ×1,000.

Lymph node biopsy revealed that the normal lymph node structure was destroyed and replaced by an abnormal diffuse infiltration of lymphocytes. Immunohistochemical staining revealed that the infiltrated lymphocytes were CD43, CD123 and CD68-positive (Fig. 2), a small scattering of cells were positive for CD20 and 40% of cells were Ki-67-positive. However, the cells were found to be CD10 and terminal deoxynucleotidyl transferase (TdT)-negative. Bone marrow aspiration analysis demonstrated that the blast cells consisted of 52% mononuclear cells and had variable sizes, agranular cytoplasm and irregular nuclei (Fig. 3). Bone marrow biopsy revealed hypercellularity with diffuse infiltration of tumor cells, accompanied by evident hyperplasia of fibrous tissue. Flow cytometric analysis of the bone marrow revealed the following cellular characteristics: CD4^+^, CD56^+^, CD117^+^, CD33^+^, HLA-DR^+^, CD43^+^, CD123^+^ (Fig. 4), CD34^−^, MPO^−^, CD36^−^, CD64^−^, CD303^−^, CD304^−^, CD19^−^, CD10^−^, CD20^−^, CD38^−^, CD138^−^, CD13^−^ and TdT^−^. Traditional R-banding cytogenetic analysis did not detect any further chromosome abnormalities. The marrow sample was further probed using split-signal fluorescence *in situ* hybridization (FISH), which identified the rearrangement of ETV6 (Fig. 5). T-cell receptor gene rearrangement was not detected. Diagnosis of BPDCN was conclusive, based on the aforementioned findings. Notably, the blast cells were found to be positive for CD33 and CD117, which are myeloid leukemia-associated antigens.

Initially, the patient was treated with the VDCP regimen (vincristine: 2 mg, i.v. drip, days 1, 8, 15 and 22; daunorubicin: 40 mg, i.v. drip, days 1–3 and 15–17; cyclophosphamide: 1 g, i.v. drip, days 1 and 15; and prednisone: 60 mg, p.o., days 1–14, decrement from day 15) (6). Following the intensive chemotherapy treatment, the proportion of tumor cells decreased from 52 to 3.5%. The patient suffered from severe headache in the complete remission stage; however, brain CT scans showed no significant abnormalities. Subsequently, several lumbar punctures and intrathecal chemotherapy (cytarabine, methotrexate and dexamethasone) were performed, and the patient recovered gradually. However, three weeks after the treatment period, the tumor cells rapidly increased. Thus, the patient was treated with the MEVP regimen (mitoxantrone: 10 mg, i.v. drip, days 1–3, etoposide: 100 mg, i.v. drip, days 1–5; vincristine: 2 mg, i.v. drip, days 1 and 8; and prednisone 60 mg, p.o., days 1–14) (6), followed by the HAD regimen (homoharringtonine: 3 mg, i.v. drip, days 1–5; cytarabine: 150 mg, i.v. drip, days 1–5; and daunorubicin: 40 mg, i.v. drip, days 1–3) (7), according to the immune phenotype (CD33+, CD117+ and HLA-DR+). The treatment had no effect on the tumor growth and the patient succumbed to severe pulmonary infection seven months after the diagnosis. The present study was approved by the Ethics Committee of the Binzhou Medical University Hospital. Informed consent was obtained from the patient’s family prior to participation in the current study.

## Discussion

BPDCN is a rare and highly aggressive hematologic malignancy that involves plasmacytoid dendritic cell precursors. The nomenclature of this disease has evolved over time. In 1994, the disease was first identified as blastic NK cell lymphoma/leukemia by Adachi *et al* (5). In 2005, the WHO-European Organization for Research and Treatment of Cancer identified the disease as a CD4^+^/CD56^+^ hematodermic neoplasm due to its derivation from a plasmacytoid dendritic cell precursor (4). The term BPDCN was enlisted in the 2008 WHO classification of hematopoietic and lymphoid tissue tumors (1). BPDCN frequently involves the skin, lymph nodes, peripheral blood and bone marrow. Histopathologically, BPDCN is characterized by a diffuse, monomorphous infiltrate of medium-sized blastic cells with irregular nuclei, fine chromatin and one-to-several small nucleoli (5). The first manifestation of the disease is often cutaneous involvement. The majority of patients present skin involvement, while a number of cases with only skin involvement have been reported (8). The present study presents a rare case of leukemic manifestation with lack of skin involvement. To date, few such cases have been studied worldwide (9). To the best of our knowledge, the present study is the first reported case of BPDCN with leukemic involvement and lack of skin involvement in China. Notably, the possibility of central nervous system involvement during the development of BPDCN was observed. Therefore, prevention of central nervous system leukemia in patients suffering from the reported BPDCN type is necessary in the complete remission stage.

BPDCN is characterized by a medium-sized, dense monomorphous infiltration with a blastoid morphology (5), typically expressing CD4, CD56, CD43, CD45RA, CD123, BDCA-2/CD303 and TCL1 antigens. The expression of CD56 has been rarely found to be negative. In addition, BPDCNs usually lack most myeloid antigens; thus, CD117 and CD33-positve BPDCNs are extremely rare. The expression of CD68 is normally negative in BPDCNs, whereas a positive CD68 expression indicates that the BPDCN may be transformed into acute or chromic leukemia and particularly monocytic leukemia. In the present case, lymph node immunohistochemical staining revealed a small scattering of cells positive for CD68, which may be one of the reasons for the leukemic transformation.

ETS variant gene 6 (ETV6), mapped to 12p13, is an ETS family transcription factor that is essential in hematopoietic processes (3). ETV6 gene-involved chromosomal translocations have been detected in numerous hematological malignancies characterized by fusion with a number of partner genes. ETV6 mainly codes for tyrosine kinases or transcription factors, which are important in the initiation, progress and prognosis of a disease. In the present study, split-signal FISH was used and ETV6 rearrangement was observed. To the best of our knowledge, this is the first case of ETV6 involvement in translocation in BPDCN. Repression of ETV6 appears to be important in the regulation of cell growth and differentiation (10,11). In addition, ETV6 has been shown to stimulate erythroid differentiation of a murine erythroid leukemia cell line (11). However, further research is required to establish whether ETV6 rearrangement participates in the pathogenesis and leukemic transformation of BPDCN. Furthermore, BPDCN usually has an abnormal and complex karyotype, lacking specific chromosomal aberrations (12). ETV6 rearrangement may not be the primary transforming event in BPDCN, while the abnormal karyotype observed in the patient of the present study may be exceptional in BPDCN. However, the molecular pathogenesis of the disease remains unclear.

BPDCN is not sensitive to conventional chemotherapy and the prognosis is poor. Currently, no standard treatment for BPDCN exists. However, AML chemotherapy regimes may not be the optimal treatment methods for BPDCN. By contrast, acute lymphoblastic leukemia (ALL) protocols have been shown to be more advantageous (13). In the present study, the patient received ALL-like schemes (VDCP, MEVP) and AML-like schemes (HAD) in succession. A complete remission was achieved in the early stage; however, the two schemes were invalid in the relapse phase. Allogeneic hematopoietic stem cell transplantation should be considered to be the most effective treatment measure.

In conclusion, BPDCN is a rare disease with a poor prognosis. It is similar to acute leukemia but with high-risk features. The current study is the first, to the best of our knowledge, to describe the ETV6 rearrangement in BPDCN. The significance of the ETV6 rearrangement in BPDCN requires further study. Additional studies with larger sample sizes are required in order to increase understanding of the disease and its molecular and biological features.
